# Identification of evolutionarily meaningful information within the mammalian RNA editing landscape

**DOI:** 10.1186/gb4157

**Published:** 2014-01-28

**Authors:** Yiannis A Savva, Robert A Reenan

**Affiliations:** 1 Department of Molecular Biology, Cell Biology and Biochemistry, Brown University, 185 Meeting St, Providence, RI 02912, USA

## Abstract

A large comparative genomic sequence study has determined the extent of conservation between RNA editing sites within the mammalian evolutionary tree.

See related research by Pinto et al., http://genomebiology.com/2014/15/1/R5

## Expansion of the RNA editing universe

**Figure 1:**
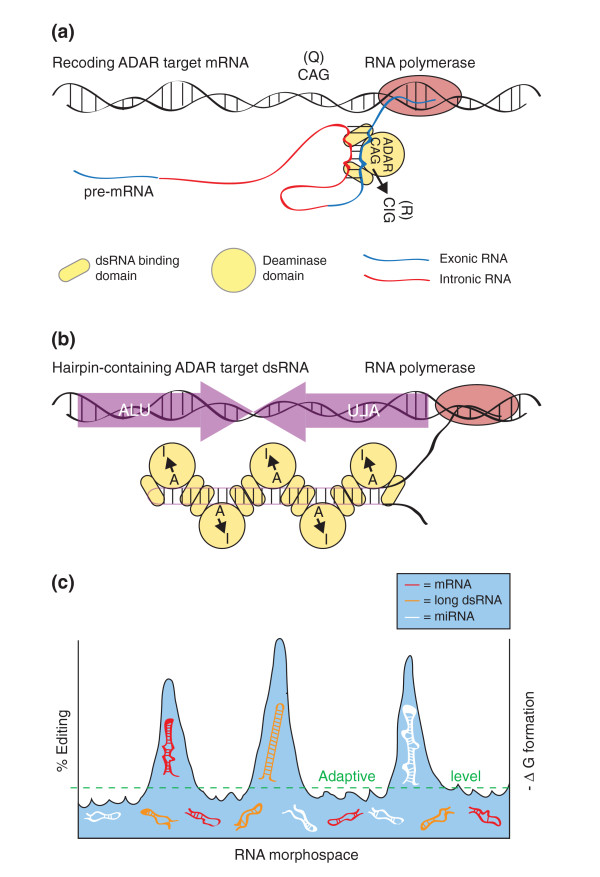
**An overview of RNA editing. (a)** Specific RNA editing occurring in a pre-mRNA. The nascent transcript folds into a complex dsRNA structure, pairing coding sequences (blue) with highly conserved intronic sequences. Structural features (for example, bulges and loops) in the duplex region focus ADAR’s deaminase activity on a few or one adenosine residue. **(b)** Promiscuous editing of long repeat dsRNAs. Shown here is an inverted repeat of Alu elements whose transcription produces a long nearly perfect dsRNA. Numerous ADAR editing events can occur in such a substrate, altering its structure, and potentially interfering with downstream processes (for example, Dicer processing into endo-siRNAs). **(c)** A hypothetical scheme for conserved versus less-conserved RNA editing events. All RNA molecules can potentially form secondary structures, and thus potentially bind and be acted upon by ADAR. Those that are minimally structured will be edited poorly or not at all. The vast majority of RNAs will fall into this category, and be well below the action of serving as a variant incipient adaptation acted upon by natural selection (below dashed green line). Certain RNAs will more readily serve as ADAR targets, and will as a consequence also possess more dsRNA character. Such events can be acted upon by natural selection and serve as sources of variation in the expression of RNAs (for example, mRNAs, long non-coding dsRNA, miRNA precursors). Natural selection will preserve certain structural features, such as those in pre-mRNA and miRNA precursors, to ensure highly evolved edited structures. Other long dsRNAs, such as inverted repeat hairpin dsRNAs, will be edited as part of the regulation of host defenses to viruses and selfish genetic elements. ADAR, adenosine deaminase acting on RNA; dsRNA, double-stranded RNA; miRNA, microRNA, siRNA, small interfering RNA.

Generating cellular proteomes relies on the faithful decoding of genetic information. A complex network of cellular machines transcribes DNA into matured processed mRNA that is then translated into protein products, which are used by the cells to carry out basic biological functions. It is widely accepted that organismal complexity arises via the expansion of the genetic information potential by post-transcriptional modifications, such as alternative splicing and RNA editing. The latter is mediated in metazoans by a highly conserved protein family known as adenosine deaminase acting on RNA (ADAR) [1]. These enzymes hydrolytically deaminate adenosines to inosines (A-to-I) in double-stranded RNA (dsRNA) substrates. A-to-I RNA editing generates subtly different protein products by altering the primary sequence of target genes, since upon translation the ribosomal machinery interprets inosines as guanosines, leading to A → G substitutions (Figure 1a). Therefore, electropherograms derived from edited cDNAs include A/G mixed peaks, which are considered to be a hallmark of A-to-I RNA editing. A decade ago, only few editing sites had been known to exist due to their accidental discovery by comparison of cDNA to genomic DNA sequences. However, a comparative genomics approach in *Drosophila melanogaster* uncovered a phylogenetic signature of RNA editing [2]. Since ADAR-mediated editing ensues through the formation of highly structured and frequently complex dsRNA substrates, necessary *cis*-regulatory elements should be highly conserved across *Drosophila* species. Indeed, *cis*-elements that promote imperfect dsRNA formation in pre-mRNA, also known as editing complementary sequences (ECS), usually found in introns, are highly conserved (Figure 1a). It is generally thought that the nature of the structural imperfections in these dsRNAs lend the specificity in determining which adenosines are edited by ADAR. In addition, sequence conservation is higher in exonic sequences near editing sites when compared with adjacent exons due to the functional constraints imposed by RNA structure. Using this pattern of evolutionary conservation led to the discovery of approximately 50 new editing sites in 16 different genes. Intriguingly, the conserved editing sites were found to be present in genes encoding proteins that are involved in electrical and chemical neurotransmission, including synaptic release proteins and voltage-gated and ligand-gated ion channels. To further understand the biological significance of RNA editing, contemporary studies use deep sequencing technologies to identify novel RNA editing sites. During the last decade, deep sequencing analysis rapidly expanded the RNA editing landscapes in various organisms, including humans, mouse and *Drosophila*. However, the extent of evolutionary conservation between the numerous mammalian RNA editing sites across the evolutionary tree is currently unknown.

## The RNA editing enigma

RNA editing sites are scattered within the human, mouse and *Drosophila* transcriptomes. Geographically, editing sites are found in both coding regions (exons) and in non-coding regions (5′ UTRs, 3′ UTRs, introns and intergenic) throughout genomes. Another theme from the deep-sequencing era is that much of the informational content of genomes is transcribed into non-coding RNA, whose functions largely remain to be determined. Comparison of RNA editing landscapes between different genetic model organisms has uncovered diverse ADAR substrates and revealed discrepancies within RNA editing systems. While the number of exonic editing sites that lead to non-synonymous amino acid substitutions (genomic recoding) varies significantly across a broad range of organisms, a common theme seems to be emerging. The majority of RNA editing sites are located in non-coding regions within genomes. For example, in the human genome, RNA editing sites are highly over-represented in *Alu* elements restricted within introns of transcribed genes [3]. *Alu* elements are lineage-specific repeat genomic sequences, which participate in dsRNA template formation acting as ADAR substrates (Figure 1b). Unlike mRNA targets of ADAR, such more perfect and extensive dsRNA substrates lend themselves to more extensive deamination, with up to 40% of adenosines in the duplex region undergoing modification. Similarly, within the mouse genome the majority of RNA editing sites are found in B1 short interspersed element repeats. RNA editing in non-coding regions of a transcript can have several functional consequences such as: creation or elimination of splicing signals, exonization of repeat elements, nuclear retention, regulation of microRNA (miRNA) biogenesis and function, cellular defense, and regulation of RNA interference [4]. It is thought that the collection of RNA editing sites in an organism’s transcriptome contributes equally to the appropriate functioning of the nervous system, as exemplified from the generation of ADAR deficiencies in various genetic models [5]. In *Drosophila*, deletion of the *adar* locus results in severe neurological phenotypes, including extreme uncoordination, seizures and neurodegeneration. Furthermore, mice homozygous for ADAR1 null mutations die during early development due to severe apoptosis. In addition, ADAR2 null mutant mice experience repeated seizure episodes and die soon after birth. Interestingly, the lethality phenotype of the ADAR2 null mice is rescued by the introduction of the edited allele of a single RNA editing site in the glutamate receptor channel, GluR-B Q/R site [6]. This observation suggests that certain RNA editing sites are more physiologically critical compared with others existing in the same genome. Thus, a general enigma in ADAR-mediated editing is exactly how to determine which, of many, RNA editing sites are functionally important, and which may have no discernable function.

## A unique set of RNA editing sites

In order to identify functionally important RNA editing sites from a vast RNA editing landscape, a recent study by Pinto *et al*. [7] used evolution as a key discriminator to delineate highly conserved sites in mammalian lineages. Taking advantage of enormous RNA-seq datasets of both human and mouse transcriptomes, the authors applied a standard BLAST alignment tool to compare 40 base pairs upstream and downstream of human genomic sequences surrounding an RNA editing site to the mouse genome. A set of basic filters was applied to the datasets to retain only RNA editing sites located at the same exact position in both human and mouse genomes. Surprisingly, this simple procedure identified 59 highly conserved editing sites. This set of RNA editing sites, which were termed evolutionary selected sites (ESS), represents 0.004% of the known human editing sites to date. Furthermore, the authors demonstrated that the percentage of ESS sites does not increase as RNA-seq data accumulate. Using a small fraction of the available RNA-seq data sets from 15 different mouse strains, the authors were able to retrieve approximately 95% of the ESS sites in any random choice of two mouse strains. While the ESS sites were found across all the mouse strains tested, the non-conserved sites were not consistently detected, suggesting that the ESS set is exquisitely specific. In addition, evidence for the presence of ESS sites can be found in RNA-seq data from an additional four genomes within the mammalian evolutionary tree: rat, cow, opossum and platypus. The presence of ESS sites across large evolutionary distances suggests a functionally important role in mammalian biology for these sites, despite the fact that the set is surprisingly small.

## The nature of functionally important RNA editing sites

Due to the degeneracy of the genetic code, RNA editing can cause both synonymous and non-synonymous changes. In *Drosophila*, the majority of highly conserved RNA editing sites lead to non-synonymous amino acid changes in functionally important and highly conserved residues within proteins. Similarly, the majority of the mammalian ESS editing sites lead to amino acid recoding. Specifically, 37 out of 59 ESS editing sites are found in coding regions of the genome and 35 of them lead to non-synonymous amino acid substitutions (94%). In addition, most of the non-coding ESS editing sites (22/59) occur in transcripts of genes that are also edited elsewhere in their coding sequence. Recent studies in *Drosophila* identified editing sites in ECS non-coding genomic elements [8,9]. Not surprisingly, using structural RNA prediction software (mfold), Pinto *et al.* showed that most of the ESS editing sites in non-coding regions are located in potential ECS elements. Furthermore, two of the ESS editing sites are found in miRNAs in agreement with the previously described regulation of miRNA biogenesis and function through RNA editing. Further analysis revealed certain features of the ESS editing set. First, the ESS editing sites display higher levels of expression when compared with the non-conserved editing sites set. Second, the ESS editing set exhibits higher levels of editing and, more importantly, these levels of editing display striking conservation across 15 mice strains and between human and mouse. These observations indicate that the editing levels of the conserved mammalian RNA editing sites are set within the evolutionary tree and that the precise ratios of edited/non-edited repertoires of protein products may contribute to the optimization of cellular physiology. Lastly, similar to the highly conserved RNA editing sites reported in *Drosophila*, the ESS editing sites are overrepresented in genes that play a pivotal role in nervous system functions such as synaptic release and ion transport. This observation suggests that natural selection acts through RNA editing to evolve RNA structures that are acted upon by ADARs to genetically recode the proteome associated with neurotransmission and thereby fine-tune brain physiology.

## Unweaving the roles of RNA editing

Abnormalities in the RNA editing pathway are associated with multiple nervous system disorders including schizophrenia, epilepsy, suicidal depression and amyotrophic lateral sclerosis [10]. The conserved RNA editing sites within the mammalian evolutionary tree reported by Pinto *et al*. [7] have the potential to contribute to a better understanding of the link between the RNA editing process and various neurological diseases. With the rapid advent of genetic engineering techniques, specific RNA editing sites could be precisely examined *in vivo* in various animal models, thus uncovering functions of ESS single RNA editing events. Furthermore, the specific characteristics of the non-conserved set of RNA editing sites prompted Pinto *et al*. to propose that these events are simply a consequence of overactivity of RNA editing enzymes with no apparent evolutionary value. Certainly, particular specific RNA editing events are under intense selective pressure, yet all RNAs are structured to some degree due to the single-stranded nature of RNA. Many RNAs, from all classes, probably serve as poor ADAR substrates (Figure 1c). Low-level editing of barely structured RNAs is not likely to be under intense scrutiny by natural selection, but sequence variants that become more structured, or obtain more stable structures under altered environmental conditions, may be better ADAR substrates, and then be vetted by natural selection for their adaptive consequences. Further sequence drift, under selective conditions, could shape RNAs into efficient ADAR substrates over generations. Other targets, for instance long dsRNA transcribed from recent duplication or transposition events, may immediately be hyperedited, leading to intersection between RNA editing and small RNA processing.

Multiple reports suggest that the RNA editing pathway is highly sensitive to external and internal stimuli such as temperature and inflammation. Therefore, future studies should aim to investigate how these two different sets of mammalian RNA editing sites respond to alterations of environmental stimuli. Non-conserved RNA editing events within mammalian lineages could represent variations in how RNA editing reshapes a specific transcriptome/proteome in response to external or internal changes in the environment. Such editing sites could represent derived characters, as such, which could then be interpreted as molecular adaptations in cellular functions. Studies such as that by Pinto *et al*. open the doorway for identifying both the conserved and, perhaps equally interesting, non-conserved species-specific RNA editing events that have shaped, and been shaped by, evolution.

## Abbreviations

ADAR: Adenosine deaminase acting on RNA; A-to-I: Adenosine to inosine; cDNA: complementary DNA; dsRNA: double-stranded RNA; ECS: Editing complementary sequence; ESS: Evolutionary selected sites; miRNA: microRNA.

## Competing interests

The authors declare that they have no competing interests.
